# The use of interleukin-23 inhibitors to treat immune-related psoriasis induced by immune checkpoint inhibitors and in patients with active cancer: A case series and review of the literature

**DOI:** 10.1177/2050313X231181035

**Published:** 2023-06-16

**Authors:** Cynthia Fournier, Marcus Otho Butler, Maxwell Benjamin Sauder

**Affiliations:** 1 Princess Margaret Cancer Centre, Toronto, ON, Canada; 2 Toronto Research Center Inc., Toronto, ON, Canada; 3 Toronto Dermatology Center, Toronto, ON, Canada

**Keywords:** Cancer, dermatology, immunology, inflammatory, dermatoses, psoriasis, immunotherapy, immune-related adverse events, melanoma

## Abstract

Immune checkpoint inhibitors have revolutionized cancer treatment but can induce immune-related adverse events including psoriasis. Managing immune-related psoriasis or psoriasis in a cancer setting is challenging with a lack of safety data. We describe three patients receiving interleukin-23 inhibitors to manage psoriasis in an active cancer setting, including one with immune-related psoriasis. Interleukin-23 inhibitors were effective for all patients. While being on interleukin-23 inhibitors, one patient had cancer partial response, one had deep partial response but then progressed and died from her melanoma, and one suffered melanoma progression.

## Introduction

Immune checkpoint inhibitors (ICIs) have revolutionized treatment of many cancers. ICIs act by releasing T lymphocytes inhibition and restoring the ability of the immune system to attack tumor cells. ICIs may induce T cells to attack normal tissue and induce immune-related adverse events (irAEs). Skin is the most common organ affected^1^ and immune-related psoriasis may occur. Managing psoriasis in an oncological setting is challenging. We describe three cases of immune-related psoriasis and psoriasis in an active cancer setting treated with interleukin-23 (IL-23) inhibitors.

## Cases

A 52-year-old male with metastatic non-small cell lung adenocarcinoma, BRAF G469A mutated with high PD-L1 expression, was enrolled in a trial and received a MEK inhibitor and pembrolizumab (Table 1). After two cycles, he developed a psoriasis flare (body surface area (BSA) = 30%, Psoriasis Area and Severity Index (PASI) = 23.1) with psoriatic arthritis. He discontinued the trial. Guselkumab (dermatology) and prednisolone 25 mg daily (rheumatology) were initiated. Two months later, BSA was 10%, PASI was 10.8, and he tapered steroids to 10 mg. His scan 3 months after terminating trial and 2 months after initiating guselkumab showed cancer progression. Chemotherapy (carboplatin and pemetrexed) with pembrolizumab was initiated. His PASI continued to decrease to 7.4; however, arthritis worsened with the need to restart systemic steroids, increase guselkumab to 100 mg every 4 weeks, and hold pembrolizumab. His PASI decreased to 5.9 at 8 months of guselkumab. After 7 months on chemotherapy and pembrolizumab, while being on guselkumab for 9 months, he had cancer partial response.

**Table 1.** Summary of clinical findings, cancer history, cancer treatment, and cancer outcome in three patients receiving IL-23 inhibitors to treat immune-related psoriasiform eruption induced by immune checkpoint inhibitors or in a cancer setting.

Patient	Sex	Age	Cancer	Cancer treatment	Psoriasis subtype	History of psoriasis before cancer treatment	Prior psoriasis treatment	Biologic	Response to biologic	Time of follow-up after the beginning of IL-23 inhibitor	Cancer outcome
1	Male	52	Stage IV non-small cell lung adenocarcinoma	- MEK inhibitor (binimetinib) and pembrolizumab - carboplatin, pemetrexed and pembrolizumab	Plaque-type psoriasis with psoriatic arthritis	Yes	- Enstilar foam - prednisolone	Guselkumab 100 mg week 0, week 4, and every 8 weeks. Increased to 100 mg every 4 weeks to manage psoriatic arthritis.	Good response for the skin, partial response for the joints	9 months. After 9 months, IL-23 was discontinued and switched to methotrexate for the joints	Partial response
2	Female	58	Metastatic melanoma	- dabrafenib and trametinib - pembrolizumab - encorafenib and binimetinib - radiation therapy	Plaque-type psoriasis with psoriatic arthritis	Yes	- Enstilar foam - tacrolimus ointment - clobetasol ointment	Guselkumab 100 mg week 0, week 4, and every 8 weeks	Excellent response for the skin, no sign of arthritis	34 months	Deep partial response for 2 years, then progression of disease and death
3	Male	60	Metastatic melanoma	- ipilimumab and nivolumab - nivolumab - dabrafenib and trametinib - surgery - radiation therapy	Guttate psoriasis, then plaque-type psoriasis, palmoplantar pustulosis, and psoriatic arthritis	Yes, and positive family history (sister)	- Enstilar foam	Risankizumab 150 mg week 0, week 4, and every 12 weeks. Increased to 150 mg every 6 weeks after 2 months of treatment.	Excellent response for plaque-type psoriasis, good response for palmoplantar pustulosis	5 months	Brain metastases progression

IL, interleukin; IV: intravenous.

A 58-year-old female received first-line targeted therapy (dabrafenib/trametinib) for 2 years complicated by pyrexia and psoriasis flares for BRAF-mutated metastatic melanoma (Table 1). Due to medication intolerability and cancer progression, plan was to switch to immunotherapy. Medical oncology required her psoriasis under control. Guselkumab was initiated followed by pembrolizumab 2 months later. PASI 90 was achieved after four guselkumab injections. Throughout 2 years of treatment, she achieved a melanoma deep partial response and without psoriasis flares with BSA below 1%. She subsequently developed immune-related vitiligo and alopecia areata. At 2 years on pembrolizumab and guselkumab, she suffered melanoma oligoprogression. It was decided to hold guselkumab, continue pembrolizumab, and give radiation. Her melanoma continued to progress, pembrolizumab was discontinued, and she started encorafenib/binimetinib. Six months after guselkumab discontinuation, her plaque-type psoriasis and psoriatic arthritis flared. Guselkumab was restarted. After one injection, her psoriasis significantly improved. She experienced severe hand foot skin reaction and retinopathy induced by targeted therapy. Cancer treatment was discontinued and she succumbed to melanoma.

A 60-year-old smoker man was diagnosed with metastatic BRAF-mutated melanoma (Table 1). He received radiation to a brain metastasis and two cycles of combination immunotherapy (ipilimumab/nivolumab) complicated by colitis and arthritis. He had lymph node complete response and brain metastasis partial response. Colitis and arthritis were managed using high-dose systemic steroids, infliximab, vedolizumab, hydroxychloroquine, and sulfasalazine. He was rechallenged with single agent nivolumab 6 months later but experienced a severe colitis flare after one cycle and immunotherapy was permanently discontinued. Later, he suffered brain metastasis progression managed with surgery and radiation. Adjuvant targeted therapy (dabrafenib/trametinib) was started 9 months later. Three months into targeted therapy, he developed guttate psoriasis. He was managed using topicals, but he then developed severe plaque-type psoriasis (PASI 16.2) with palmoplantar pustulosis. Infliximab was discontinued and risankizumab was started. After two injections, he achieved a PASI 75. Palmoplantar involvement partially improved, so risankizumab was increased to every 6 weeks 4 months into treatment. Two months later, palmoplantar pustulosis significantly improved. He did not experience any colitis flare. After 5 months on risankizumab, he suffered brain metastases progression.

## Discussion

ICIs are increasingly used to treat many cancers. Since the mechanism of action is releasing the inhibitory brakes on T-lymphocytes, ICIs can activate T-cells to attack normal tissue and induce irAEs. Immune-related psoriasis can be a flare of pre-existing psoriasis or the new onset. Approved IL-23 inhibitors for psoriasis include guselkumab, risankizumab, and tildrakizumab. There is no contraindication to use them in a cancer setting according to monographs. A study evaluating safety with up to 5 years of treatment with guselkumab did not show increased risk of malignancy.^2^ Safety studies of risankizumab showed that cancer risk was like the one associated with psoriasis.^3^

There are limited data on the biologics use in cancer patients. In 17 phase II and III risankizumab trials, 10 patients with a malignancy history were included; eight remained in remission and two experienced breast cancer recurrence.^3^ Guselkumab was used in seven patients with low-grade cancer history in remission, but without cancer outcome discussion.^4^ One patient received guselkumab for plaque psoriasis and developed lung adenocarcinoma.^5^ Guselkumab was held and he received chemotherapy. Guselkumab was restarted for psoriasis flare. No lung cancer relapse was observed with a 3-month follow-up.

There is a lack of evidence regarding biologics to treat cutaneous irAEs. One patient with stage IIIC lung cancer who developed psoriasis, nail psoriasis, and psoriatic arthritis induced by durvalumab discontinued ICIs and received guselkumab.^6^ Guselkumab was effective, durvalumab was not rechallenged, and there was no cancer recurrence. One patient with stage IIIB melanoma on adjuvant nivolumab developed psoriasis and was treated with risankizumab.^7^ Nivolumab was permanently discontinued and the patient had a durable psoriasis response. Melanoma remained in remission 1 year into risankizumab.

We report three patients with active malignancies treated with IL-23 inhibitors. Patient 1 experienced typical immune-related psoriasis. Patient 2 experienced psoriasis flare before receiving immunotherapy while being on targeted therapy. Psoriasis triggered by targeted therapy has been described.^8,9^ Patient 3 likely experienced delayed immune-related psoriasis. irAEs can occur up to 1 year after the last immunotherapy infusion. Although, other etiologies cannot be excluded and include TNF-alpha inhibitor-induced paradoxical psoriasis and psoriasis triggered by hydroxychloroquine or BRAF/MEK inhibitors.

Management of moderate-to-severe immunotherapy-related psoriasis is challenging, same as managing psoriasis in a cancer setting. There is a lack of effective, safe, and evidence-based treatment options and guidelines. Topical treatments and phototherapy can be effective without affecting immunotherapy or cancer outcome. Acitretin, by its lack of immunosuppressive effects, is safe. Low-dose methotrexate is controversial. It was associated with a higher risk of squamous cell carcinoma^10^ and potentially melanoma^11^; yet these data cannot be extrapolated to active cancer. On the other hand, high-dose methotrexate is used as chemotherapy to treat some cancers. Use of immunosuppressive medications such as cyclosporine and systemic steroids is controversial as they could potentially compromise ICIs efficacy.

IL-23 inhibitors, by being immunomodulator instead of immunosuppressive, may be a safe option to treat psoriasis in an active cancer setting and for moderate-to-severe irAEs. There is a need for further research with prospective trials evaluating IL-23 inhibitors in an active cancer setting and in the setting of immune-related psoriasis.
